# Supplementary material for: Understanding Protective Factors for Men at Risk of Suicide Using the CHIME Framework: The Primacy of Relational Connectedness
Source: Int J Environ Res Public Health. 2023 Jan 27;20(3):2259. doi: 10.3390/ijerph20032259 (PMC9914937; doi:10.3390/ijerph20032259)
Supplement: Supplementary file 1 [file ijerph-20-02259-s001.zip › ijerph-2142778-supplementary.pdf]

Supplementary: Interview Guideline Questions

MAIN QUESTION	Primary Prompt/s	Secondary Prompt/s
**1. Can you please tell me about your experience/s?**	**Can you share your experience of living with thoughts of wanting to end your life?**	If the participant talks about the thoughts being unpleasant in some way, follow up with 'How do you cope with that?'
	**How did/do you experience your thoughts of wanting to end your life?**	How have these thoughts impacted your life?
		How often did/do you experience these thoughts?
	**Could you tell me about the time or times in your life when you experienced thoughts and feelings of wanting to end your life?**	What happened in the lead up to these experiences?
		What happened during?
		What happened afterwards?
		What was going on in your life at the time?<br>What was with situation in your life?
		If having difficulty add restriction on time<br>Think of your last experience?<br>Think of your most distressing experience?
	**Please describe how you were feeling during your experience(s)**	*If having difficulty add restriction on time*<br>Think of your last experience?<br>Think of your most distressing experience?
		How long did you feel this way? How long did you have these thoughts?

		Were there things that other people in your life noticed?
		What sensations do you notice in your body when you are experiencing thoughts of ending your life?

MAIN QUESTION	Primary Prompt/s	Secondary Prompt/s
**2. What has supported you through these experiences?**	**What did you find you could do to support yourself?**	What helped? [person, pet, place, thing]
	**How did you get through or manage your thoughts of ending your life?**	
	**What strategies did you use to help you get through?**	What worked and what did not work?
		Were there any steps you thought about taking, but chose not to? (e.g., reaching out to family, see a GP)
		In the moment? Longer term?
	**Were there things that were more and less helpful?**	What kinds of support was not helpful?
		Why were they not helpful?
		In the moment? Longer term?
		Why was x/y/z helpful/not helpful?
	**Was there a particular person/s who was helpful?**	Please tell me what it was about that person(s) that made them helpful to you?
		Did you need to see the person face to face or were they able to help by phone, text, or other way?

		Did you need their support immediately or were you able to wait a while? How long?
	**Was there something unexpected that helped you through those times?**	
	**What did you do to get support?**	What does support look like to you?

MAIN QUESTION	Primary Prompt/s	Secondary Prompt/s
**3. What other kind of support would have been helpful at these difficult times?**	**What other kinds of support can you think of that might have been helpful?**	Emotional support?
		Tangible support (aids and services)?
		In what way would the support have been helpful?
	**Why weren't you able to get this support?**	What makes it difficult to get this support?
		What concerns do you have about seeking support? If any?
		Were the barriers internal or external?
	**Why do you think this kind of support would have been helpful?**	
	**Would formal mental health services (private/public - psychiatrist, psychologist, social worker, a mental health service)**	How do you think you would feel if you saw a mental health professional?

	**be helpful? Probe for knowledge base in this area...**	

MAIN QUESTION	Primary Prompt/s	Secondary Prompt/s
**4. If you could envisage something that would really work for you in these difficult times, what would that look like?**	**What do you think would support others during similarly difficult times?**	
	**What would seeking help look like for you?**	Thinking about your journey and experiences, at what point would you have liked to receive this support? When do you think this help would have been most useful?
	**Who, if anyone, would be involved in giving that support?**	
	**How would you like to let people know that you need support?**	
	**What do you think is missing in existing services/supports?**	What would you have liked to have been available during this time?
		Thinking about when you had to manage this yourself, or with less support than you would have liked or needed, what would have been useful?
	**Is there anything else you can think of that would help you to navigate these difficult times, if**	Where would you go first to seek support? How would you do that?

	**there were no real-world constraints?**	

MAIN QUESTION	Primary Prompt/s	Secondary Prompt/s
<u>**Concluding Question:**</u> **In what ways do you feel you have been able to share your experience in this interview?**	**Is there anything else you would like to share that we may have missed?**	Thinking about everything that we have discussed, is there anything else you would like to revisit?
	***Summarise the study goals [1] * Is there any other knowledge you think would be useful for us to have?**	What further information, if any, would you like to share that will help us meet the goals of this project?
		What do you feel this interview was not able to capture?
		How do you feel we can improve this interview for others?

[1] *"The purpose of this study was to learn more about the individuals' experiences of people who have had thoughts about ending their lives, and what kinds of support may have been helpful or not helpful during these times. This information will be used to help us design a different kind of support that might help people to navigate these feelings. Keeping in mind the purpose of the study, is there any other knowledge you think would be useful for us to have"?*
